# The Use of Statins as an Adjunctive Periodontal Disease Treatment: Systematic Review and Meta-Analysis

**DOI:** 10.3390/dj12060150

**Published:** 2024-05-21

**Authors:** Alice Rose Greethurst, Cosimo Galletti, Roberto Lo Giudice, José Nart, Cristina Vallés, Francisco Real-Voltas, Cosme Gay-Escoda, Enrico Marchetti

**Affiliations:** 1 School of Dentistry, Department of Integrated Dentistry, International University of Catalonia, Sant Cugat del Vallès, 08022 Barcelona, Spain; alice.greethurst@uic.es (A.R.G.); cgalletti@uic.es (C.G.); freal@uic.es (F.R.-V.); 2 Department of Human Pathology of Adults and Developmental Age, Messina University, 98100 Messina, Italy; 3 Department of Periodontology, International University of Catalonia, Sant Cugat del Vallès, 08022 Barcelona, Spain; josenart@uic.es (J.N.); cristinavallveg@uic.es (C.V.); 4 Oral and Maxillofacial Surgery Department, School of Dentistry, University of Barcelona, 08022 Barcelona, Spain; gayescoda@drteknon.com; 5 Department of Teknon Medical Center, IDIBELL Institute, 08022 Barcelona, Spain; 6 Department of Life, Health and Environmental Sciences, University of l’Aquila, Piazzale Salvatore Tommasi 1, 67100 Coppito, Italy; enrico.marchetti@univaq.it

**Keywords:** periodontitis, statins, clinical parameters, radiographic parameters, diabetes mellitus, smoking

## Abstract

Background: the purpose of this systematic review was to assess the clinical and radiographic effect of subgingival-administered statins as an adjunct periodontal treatment in patients with periodontitis. Methods: Electronic literature searches in Medline/PubMed and the Cochrane Library were conducted to identify all relevant articles. Eligibility was based on inclusion criteria which included Randomized Controlled Trials (RCTs) published after 2010, where the periodontal variables were assessed before and after periodontal treatment in combination with a statin administration. The risk of bias was assessed with the ROBINS-2 tool. The outcome variables were probing depth, clinical attachment level, bleeding on probing, and bone fill in systematically healthy patients, patients with type 2 diabetes, and smokers. Results: Out of 119 potentially eligible articles, 18 randomized controlled trials were included with a total of 1171 participants. The data retrieved from the meta-analysis showed the positive effect that statins have as an adjunctive periodontal disease treatment. When comparing the different types of statins, the PD reduction in the Simvastatin group was significantly higher than the Atorvastatin group at 6 months and at 9 months, while no differences between statins were found for the rest of the outcomes. Over 66% of the articles presented an overall risk of bias with some concerns, making this a limitation of this present RCT. Conclusions: The adjunct administration of statins has proven to have a positive effect on the periodontium by improving both clinical and radiographic parameters by a considerable margin.

## 1. Introduction

Periodontitis is one of the main reasons for tooth loss [1]. It is a chronic infectious, multifactorial disease of the supporting dental tissues due to periodontopathogens that are accumulated in the plaque, causing an infection. Periodontal disease is characterized by microbially associated, host-mediated inflammation of the periodontal ligament that results in the loss of periodontal attachment, and consequently of the alveolar bone [2,3,4,5]. The major cause for the initiation of this periodontal destruction is the host’s response to the infection and the periodontal microflora [2]. Its onset and progress are modulated by a variety of risk factors, such as smoking and diabetes mellitus, and it is also considered a common independent risk factor for other diseases [6,7]. The clinical presentation is influenced by the level of oral biofilm contamination. It also differs based on the age of the patient and the lesion number, distribution, severity, and location within the dental arch [4].

According to the diagnosis, the treatment approach may vary. Over the years, scientists have found it difficult to achieve a correct diagnosis and differentiate between chronic and aggressive periodontitis [4]. As a result, in the 2017 World Workshop, a single definition of periodontitis was implemented: a patient presents periodontitis if the interdental clinical attachment level is measurable in two or more non-adjacent teeth or if the buccal clinical attachment level is 3 mm or higher with pocket depth higher than 3 mm in two or more teeth [4]. Also, a new classification was developed that entailed a staging and grading system. The former relies on the standard dimensions of severity and extent of periodontitis and assesses its complexity, while the latter estimates the future progression risk and its potential health impact [4].

Once the diagnosis is established, the aim of the periodontal treatment is to resolve the inflammation and infection, arrest further tissue damage, and regenerate the lost bony structures in order to restore function and health [5,8]. There are various surgical and non-surgical treatments, but the common approach used in the treatment of periodontal disease, which consists of the control of bacterial biofilm, has not been enough to reduce the high incidence of this disease according to [9]. The subgingival debridement may fail to entirely remove the pathogens from the periodontal pockets. Therefore, its combination of eradication of pathogenic bacteria and some adjuvants, like the distribution of antimicrobial agents, is frequently considered an effective approach [2].

Systemic antimicrobial therapy as an adjunct to the treatment has been found to be effective in the treatment of periodontitis. However, the repeated use of antibiotics has led to the emergence of resistant strains of microorganisms and side effects, thus making their usage in the treatment debatable [10]. The use of local drug delivery (LDD) has become the main advantage of periodontal treatment where smaller amounts of topical agents are distributed inside the pocket, increasing the exposure and targeting microorganisms. As a result, a higher therapeutic outcome is provided [11]. This type of therapy overcomes the problems and complications associated with the use of systemic antibiotics [10].

Statins are a type of drug that was introduced in 1987 [2]. They are a form of medicine that inhibits the 3-hydroxy 3-methylglutaryl coenzyme A (HMG-CoA) reductase used to lower blood cholesterol levels in patients with hyperlipidemia and artherosclerosis [8,11,12]. They are used to reduce the risk of cardiovascular events [3]. As with every other medication, there is no effective treatment without side effects. The most frequent side effects include muscle and liver toxicity, gastrointestinal discomfort, and interactions with other drugs that the patient could be taking. However, statins have a favorable safety and efficacy profile with a low prevalence of these adverse effects [13].

According to Jeger and Dieterle [13], there are seven different types of statins that have been put to use: Simvastatin (SMV), Rosuvastatin (RSV), Atorvastatin (ATV), Lovastatin (LV), Pravastatin (PRV), Pitavastatin (PTV), and Cerivastatin (CRV). However, Cerivastatin was withdrawn from the market due to its side effects.

Statins have been seen to have a pleiotropic effect on the oral health. Apart from its effectiveness in controlling cholesterol levels, it also has anti-inflammatory, immunomodulatory, and antioxidant effects [8]. It has been demonstrated over the years that statins decrease the production of many proinflammatory cytokines and also the inhibition of monocyte recruitment, consequently modifying the inflammatory cascades and having a positive effect on the periodontium [6,14].

Apart from these effects, statins can stimulate the expression of bone anabolic factors, such as bone morphogenic-2 protein (BMP-2), and enhance the osteoblastic differentiation and production of osteoprotegerin (OPG), contributing to a bone regenerative effect on the alveolar bone [1].

Finding the correct drug that can have not only an anti-inflammatory effect but also regenerative potentials for the periodontium would be a positive step ahead to finding a suitable treatment for periodontitis. Until now, no systematic review has studied and compared the effect of statins on the periodontium on healthy patients, smokers, and patients with type 2 diabetes mellitus. For this reason, the aim of this systematic review is to assess the clinical and radiographic effects that statins have as an adjunct to periodontal therapy.

## 2. Materials and Methods

### 2.1. Search Strategy and Focused Question

The search strategy used in this systematic review was based on the Preferred Reporting Items for Systematic reviews and Meta-Analyses (PRISMA) guidelines [15] and registered in PROSPERO under the number CRD42023415958.

The clinical question was formulated according to the PICO model [16]. The question of the systematic review was “Do subgingivally delivered statins have a positive effect on the periodontium in patients with periodontitis?”, focusing on:

P—Population: subjects with periodontitis disease.

I—Intervention: adjunct use of subgingivally administered statin gel.

C—Comparison: placebo gel administration.

O—Outcome: positive clinical and radiographic effects around the treated teeth.

### 2.2. Search Strategy

Articles published and in press in the English language were electronically searched by two independent reviewers (A.R.G. and C.G.) until 30 December 2023, with no restrictions concerning dates of coverage and publication status, across the Medline/PubMed, Cochrane Library, and BioMed Central databases.

The following key words were applied for MEDLINE/PubMed combined by Boolean operators (AND, OR, and NOT):

(((((“Periodontitis/therapy”[Majr]) AND “Hydroxymethylglutaryl-CoA Reductase Inhibitors”[Mesh]) OR “Simvastatin”[Mesh]) OR “Rosuvastatin Calcium/therapeutic use”[Mesh]) AND “Atorvastatin”[Mesh]).

For searching the remaining electronic databases, the key terms used were as follows:

– Periodontitis AND simvastatin/rosuvastatin/pravastatin/lovastatin/pitavastatin/atorvastatin;
– Statins AND periodontal therapy;
– Statins AND periodontitis;
– Periodontal disease AND statins;
– Adjunctive periodontal therapy AND statins.

### 2.3. Study Selection

Title and abstract assessment were accomplished for all the records identified through the database search. Two examiners (A.R.G. and C.G.) individually selected the studies in accordance with the inclusion criteria. Consensus solved any discrepancies.

Full-text reading was performed for articles considered suitable for the present systematic review based on the following inclusion and exclusion criteria:

Inclusion criteria:

– Full text available in English;
– Articles published in 2012 and onwards;
– Randomized Controlled Trial (RCT) performed on humans;
– The use of one of the following statin gels as an adjunct to non-surgical periodontal therapy: simvastatin, rosuvastatin, pravastatin, lovastatin, pitavastatin, or atorvastatin;
– Assessment of at least the following clinical parameters: Clinical Attachment Level (CAL), Bleeding on Probing (BoP), Probing Depth (PD), and Plaque Index (PI).

Exclusion criteria:

– In vivo studies;
– Case reports studies;
– Case series studies;
– Case control studies;
– Cross-section studies;
– Clinical trial studies;
– Patients under the systemic treatment of statins;
– RCT performed on animals.

### 2.4. Data Collection and Synthesis

The following variables were recorded by two independent examiners (A.R.G. and C.G.) for each selected study: source (in Vancouver style), study design, aim of the study, participants, type of statin used, clinical and radiographic parameters assessed, results, and conclusions. Consensus solved any discrepancies.

### 2.5. Risk of Bias Assessment

The risk of bias was assessed according to the ROBINS-2 tool [17]. The following 5 domains were analyzed from the randomized controlled trials selected:

- Bias arising from the randomization process;
- Bias due to deviations from included intervention;
- Bias due to missing outcome data;
- Bias in measurement of the outcome;
- Bias in selection of the reported result.

For each item, in cases with sufficient data, the risk of bias was identified as “low” (green), hence unlikely to completely modify the results. For missing data, the risk of bias was stated as “high” (red), and consequently capable of serious adjustment of the results. In cases with insufficient information, the risk of bias was considered to be of “some concern” (yellow), casting doubt on the study results. Finally, the overall risk of bias within a trial was assessed. It was defined as “low” if all the items were defined as low, and as “high” or “unclear” if at least one of the domains was judged as high or unclear, respectively. Two examiners (A.R.G. and C.G.) assessed each study independently and selected the studies in accordance with the inclusion criteria. Consensus solved any discrepancies.

### 2.6. Statistical Analysis

All statin groups (ATV, SMV, RSV, and others) were aggregated into a unique group to be compared against a group placebo conducting an intra-studies conventional approach: Placebo vs. Statins (whichever).

Mean differences of outcomes between placebo and statin groups were estimated for each study and time point and the weighted mean difference (WMD) as the global effect measure in a random-effects model with corresponding Z statistics, *p*-values, and 95% confidence intervals.

Regarding heterogeneity analysis, Cochran’s Q test was applied. The I^2^ index was also calculated, representing the amount of between-studies variability compared to total variability.

In the second part of the analysis, each relevant statin (ATV, SMV, and RSV) was compared individually to the placebo group using the same previous methodology.

In the third part of the analysis, all four groups (placebo, ATV, SMV, and RSV) were compared, taking into account direct and indirect effects by means of a network meta-analysis. Random-effect models were conducted to analyze the reduction in outcomes using Bayesian hierarchical estimations. The normal likelihood with linear links was considered to provide WMD between groups. Relative effects for pairwise comparisons were obtained and presented in tabular form. Bayesian 95% confidence intervals were calculated. Rank probabilities were estimated, indicating the probability for each group to be the best, the second best, and so on.

The level of significance used in the analysis was 5% (α = 0.05).

## 3. Results

### 3.1. Study Selection

**Figure 1:**
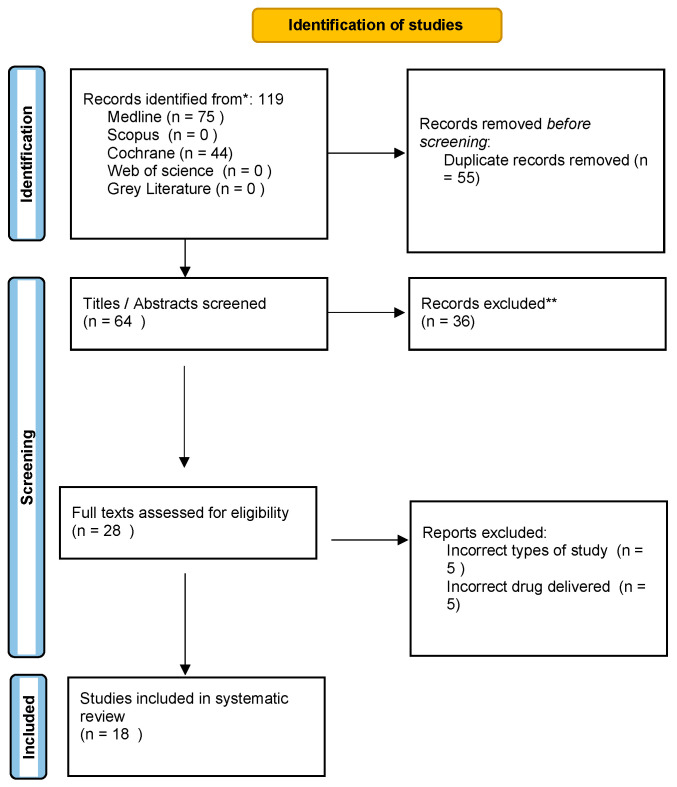
Flow chart (PRISMA format) of the screening and selection process.

The study selection process illustrated in Figure 1 shows that after a widespread electronic search, 119 articles were identified: specifically, 75 from MEDLINE/PubMed and 44 from Cochrane. Following the removal of 55 duplicates, 64 records were screened on the basis of titles and abstracts. Full-text assessment was performed on 28 articles based on the inclusion criteria, and 18 articles were finally selected for this present systematic review. All the present articles were RCTs.

Excluded studies that almost met the inclusion criteria included the use of statins on mini-flap wound healing, the comparison of oral gel to mouthwash, and the use of statins in periodontal maintenance. Two papers were excluded from the systematic review for reasons aligned with the detailed exclusion criteria outlined for the study. The first paper, titled “Effectiveness Of 1.2% Simvastatin Gel as an Adjunct to Non-Surgical Therapy in The Treatment of Chronic Periodontitis: A Split Mouth Randomized Controlled Trial”, was excluded despite being a Randomized Controlled Trial (RCT) focused on the use of Simvastatin gel in periodontal therapy. Although this study meets several inclusion criteria, such as language, publication year, and intervention type, it was ultimately excluded because it did not assess all the specified clinical parameters required by the review’s inclusion criteria, particularly failing to report on the Bleeding on Probing (BoP) [18].

Another paper, “The professional interactions between speech language therapist and dentist”, was excluded primarily because it does not pertain to a clinical trial involving the use of statin gels in non-surgical periodontal therapy. This paper instead investigates interdisciplinary communication between speech-language therapists and dentists, which is unrelated to the specific clinical focus of statins in periodontal treatment. Additionally, this study’s methodology and topic do not match the inclusion criteria focusing on RCTs assessing specific clinical outcomes in periodontal therapy using statin gels [18].

Each paper’s exclusion was justified based on adherence to the systematic review’s rigorous inclusion and exclusion criteria, ensuring that only studies meeting all specified parameters were considered for further analysis [19,20,21].

### 3.2. Study Characteristics and Descriptive Data Analysis

Each of the 18 studies comprised a minimum of 15 periodontal subjects, at least 25 years old. The majority of studies were conducted on systematically healthy patients, although two studies were carried out on patients with type 2 diabetes mellitus, and two studies focused on smokers, these being two risk factors of periodontal disease. Three groups underwent open flap debridement while the rest underwent scaling and root planing to remove calculus from the root surface with the goal of reducing the microbial levels. Simvastatin was locally delivered in seven trials, Rosuvastatin in four trials, Atorvastatin in five trials, and two trials used both Atorvastatin and Rosuvastatin in the same study.

Statin delivery was compared to a placebo group that did not use statin in all the studies. Additionally, in six studies, the intervention was also compared with other pharmacologic and non-pharmacologic interventions including metformin, alendronate, platelet-rich fibrin, and photodynamic therapy.

Clinical parameters were recorded in all the studies. The presence of plaque was recorded with the Plaque Index in all selected studies. The sulcus bleeding, recorded with the modified sulcus bleeding index (mSBI), periodontal depth (PD), and clinical attachment level (CAL), were also recorded in all the trials.

A total of 16 studies documented the infrabony defect and the defect depth reduction when analyzing the radiographic parameters.

A complete description of the selected trials, including the source, aim of the trial, drug used in the trial, follow-ups, sample, outcomes, and conclusions, is included in Table 1.

**Figure 2:**
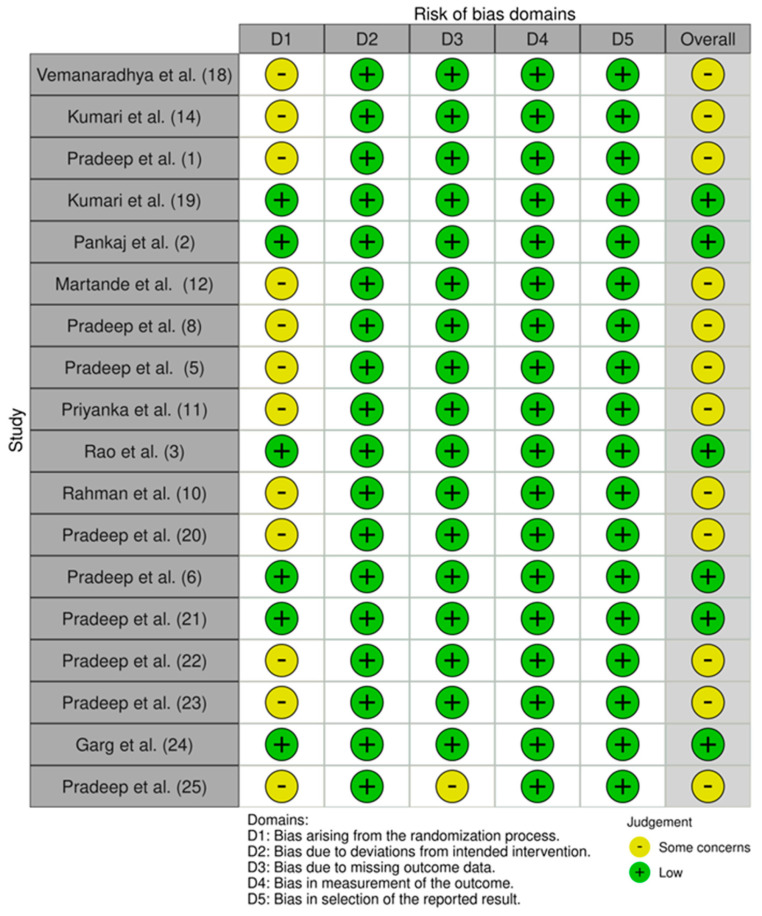
Risk of bias assessment [1,2,3,5,6,8,10,11,12,14,18,19,20,21,22,23,24,25].

Assessment of the risk of bias:

The risk of bias in the 18 randomized controlled trials was assessed with the ROBINS-2 tool reported in Figure 2. A total of 33.33% of the included RCTs presented an overall low risk of bias while the remaining RCTs presented an overall risk of bias with some concerns. The domain that caused the risk of bias to be of some concern was domain one (D1): bias arising from the randomization process. This was due to the lack of information, or due to problems in the method of sequence generation.

Statistical analysis:

The present systematic review incorporates a comprehensive statistical analysis, delineated in Figure 3, Figure 4, Figure 5, Figure 6, Figure 7, Figure 8, Figure 9, Figure 10, Figure 11, Figure 12, Figure 13, Figure 14, Figure 15 and Figure 16. Within these figures, the meta-analysis results are depicted, specifically illustrating the mean differences of all variables at the concluding time points. This representation involves comparisons between statin groups and placebo groups, as well as intra-group comparisons within each statin group.

### 3.3. Statins vs. Placebo

#### 3.3.1. Reduction in PD

9 months.

**Figure 3:**
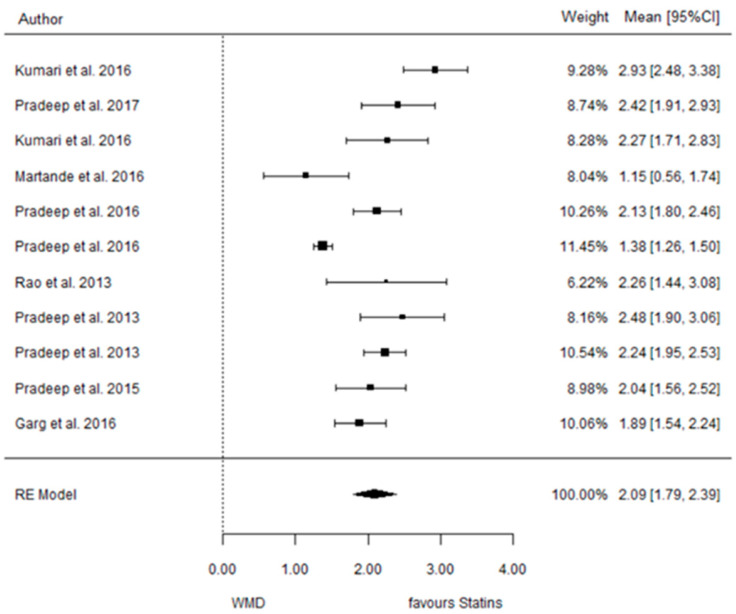
Results of meta-analysis of mean differences of PD reduction by Group at 9 months, statin vs. placebo: weighted mean difference (WMD), standard error (SE), 95% confidence interval, z test (*p*-value), I^2^ index, Cochran’s Q statistic (*p*-value) for heterogeneity, Egger’s test (*p*-value) for publication bias.

#### 3.3.2. Reduction in CAL

9 months.

**Figure 4:**
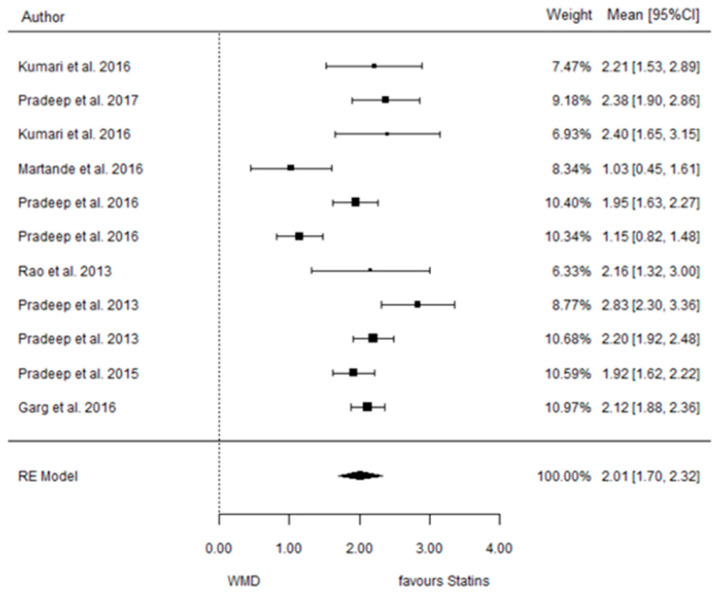
Results of meta-analysis of mean differences of CAL reduction by Group at 9 months, statin vs. placebo: weighted mean difference (WMD), standard error (SE), 95% confidence interval, z test (*p*-value), I^2^ index, Cochran’s Q statistic (*p*-value) for heterogeneity, Egger’s test (*p*-value) for publication bias.

#### 3.3.3. Reduction in IBD

9 months.

**Figure 5:**
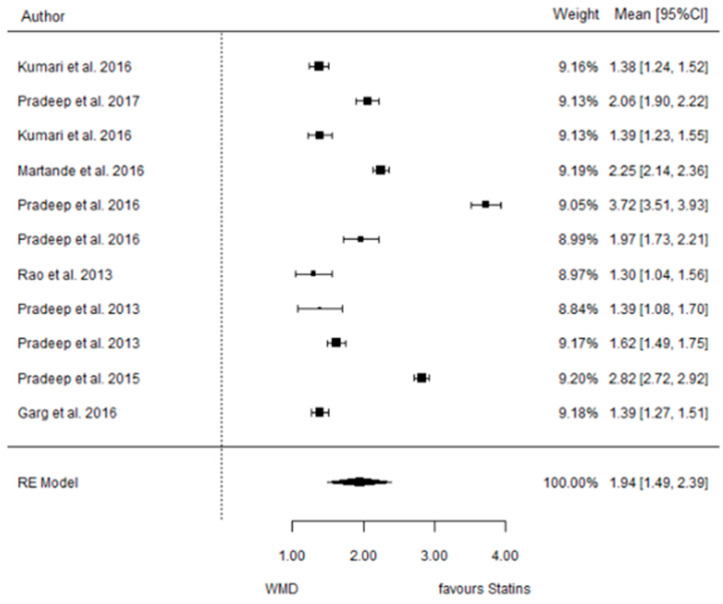
Results of meta-analysis of mean differences of IBD reduction by Group at 6 months, statin vs. placebo: weighted mean difference (WMD), standard error (SE), 95% confidence interval, z test (*p*-value), I^2^ index, Cochran’s Q statistic (*p*-value) for heterogeneity, Egger’s test (*p*-value) for publication bias.

#### 3.3.4. Reduction in RX Bone Depth

9 months.

**Figure 6:**
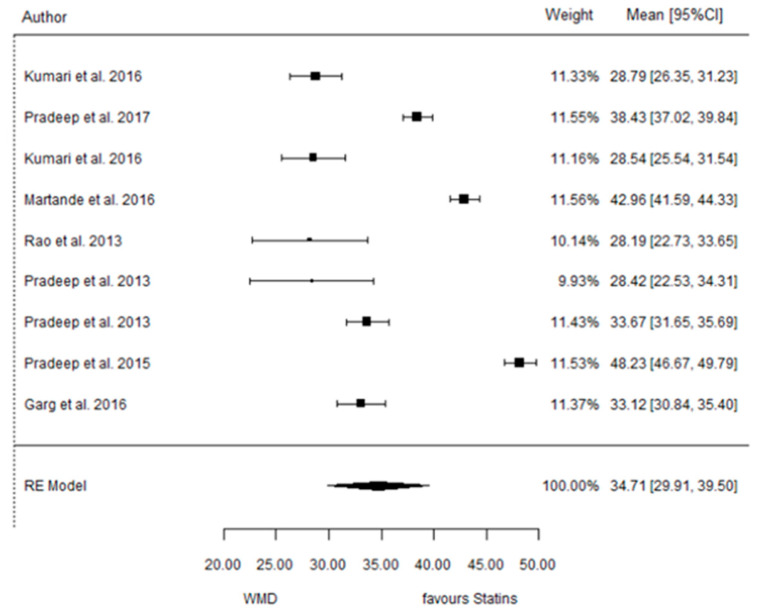
Results of meta-analysis of mean differences of Rx bone depth reduction by Group at 6 months, statin vs. placebo: weighted mean difference (WMD), standard error (SE), 95% confidence interval, z test (*p*-value), I^2^ index, Cochran’s Q statistic (*p*-value) for heterogeneity, Egger’s test (*p*-value) for publication bias.

### 3.4. ATV vs. Placebo

#### 3.4.1. Reduction in PD

9 months.

**Figure 7:**
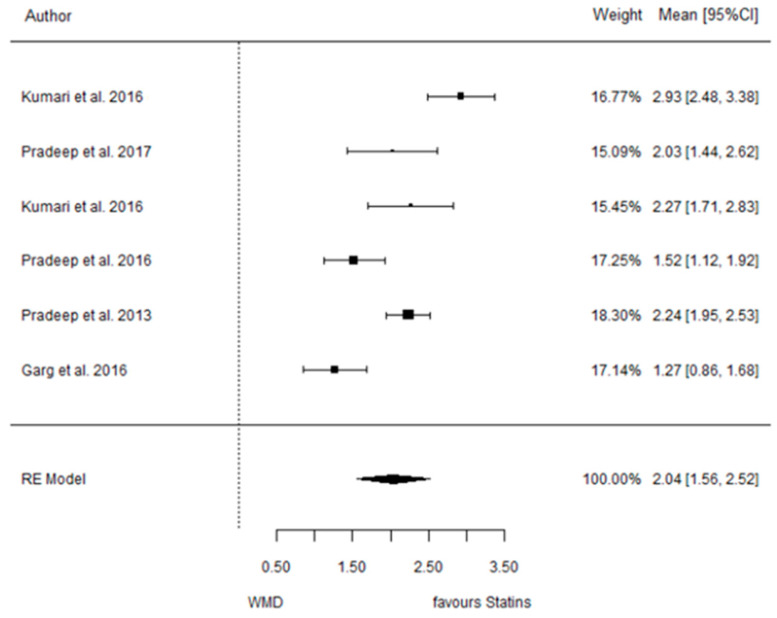
Results of meta-analysis of mean differences of PD reduction by Group at 9 months, ATV vs. placebo: weighted mean difference (WMD), standard error (SE), 95% confidence interval, z test (*p*-value), I^2^ index, Cochran’s Q statistic (*p*-value) for heterogeneity, Egger’s test (*p*-value) for publication bias.

#### 3.4.2. Reduction in CAL

9 months.

**Figure 8:**
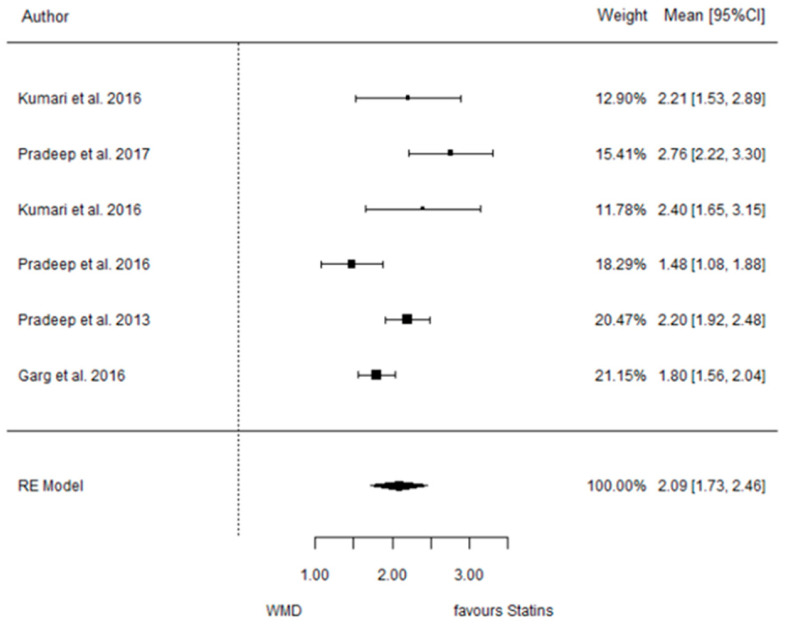
Results of meta-analysis of mean differences of CAL reduction by Group at 9 months, ATV vs. placebo: weighted mean difference (WMD), standard error (SE), 95% confidence interval, z test (*p*-value), I^2^ index, Cochran’s Q statistic (*p*-value) for heterogeneity, Egger’s test (*p*-value) for publication bias.

#### 3.4.3. Reduction in IBD

9 months.

**Figure 9:**
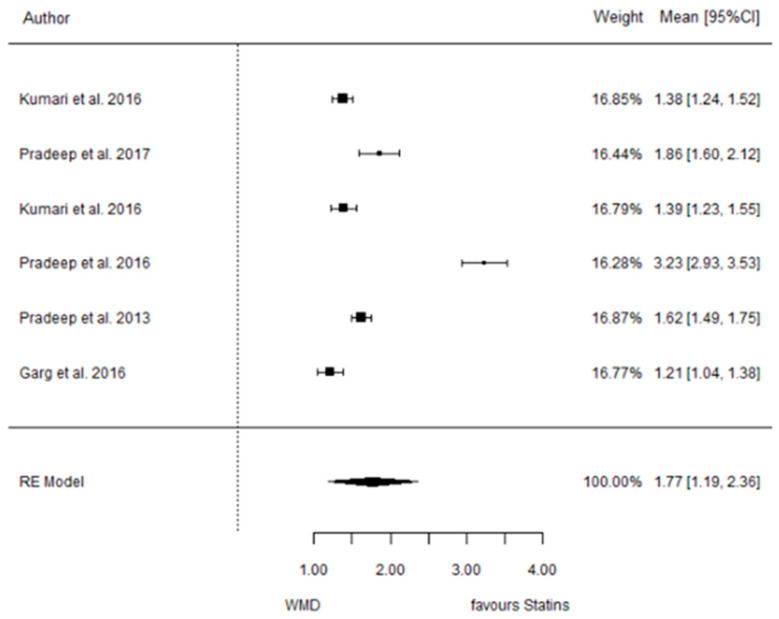
Results of meta-analysis of mean differences of IBD reduction by Group at 9 months, ATV vs. placebo: weighted mean difference (WMD), standard error (SE), 95% confidence interval, z test (*p*-value), I^2^ index, Cochran’s Q statistic (*p*-value) for heterogeneity, Egger’s test (*p*-value) for publication bias.

#### 3.4.4. Reduction in RX Bone Depth

9 months.

**Figure 10:**
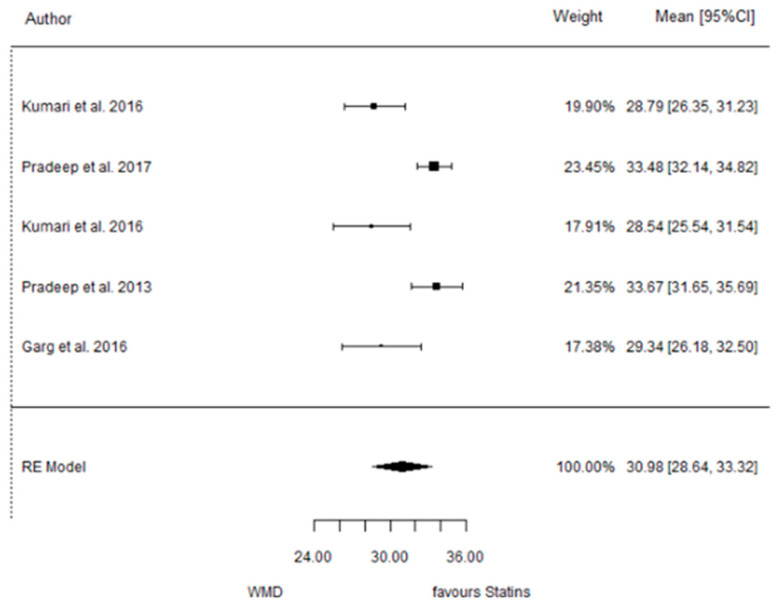
Results of meta-analysis of mean differences of Depth reduction by Group at 9 months, ATV vs. placebo: weighted mean difference (WMD), standard error (SE), 95% confidence interval, z test (*p*-value), I^2^ index, Cochran’s Q statistic (*p*-value) for heterogeneity, Egger’s test (*p*-value) for publication bias.

### 3.5. SMV vs. Placebo

#### 3.5.1. Reduction in PD

6 months.

**Figure 11:**
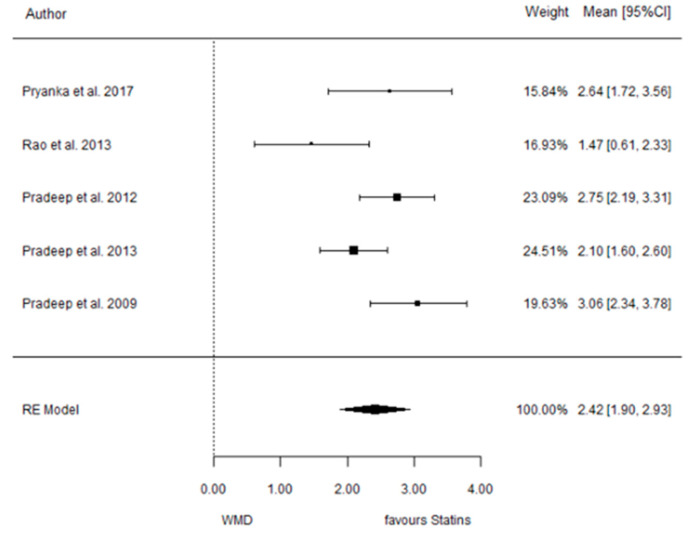
Results of meta-analysis of mean differences of PD reduction by Group at 6 months, SMV vs. placebo: weighted mean difference (WMD), standard error (SE), 95% confidence interval, z test (*p*-value), I^2^ index, Cochran’s Q statistic (*p*-value) for heterogeneity, Egger’s test (*p*-value) for publication bias.

#### 3.5.2. Reduction in CAL

6 months.

**Figure 12:**
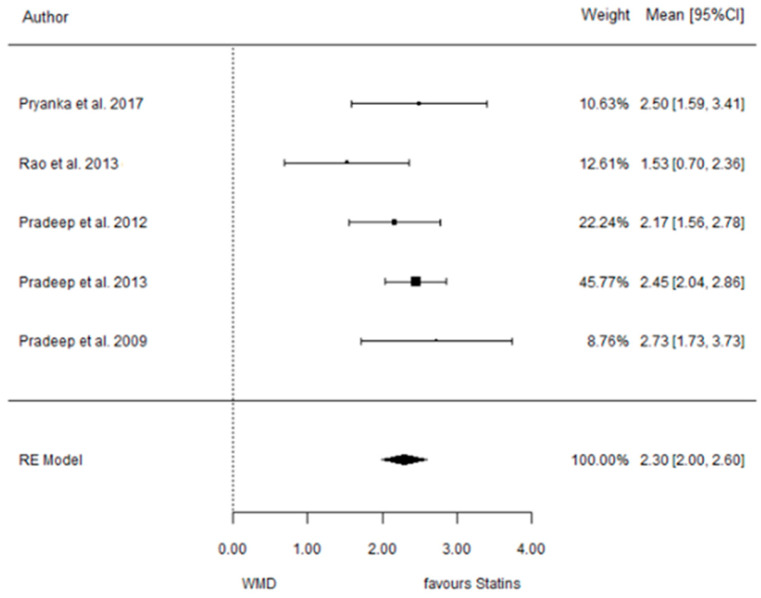
Results of meta-analysis of mean differences of CAL reduction by Group at 6 months, SMV vs. placebo: weighted mean difference (WMD), standard error (SE), 95% confidence interval, z test (*p*-value), I^2^ index, Cochran’s Q statistic (*p*-value) for heterogeneity, Egger’s test (*p*-value) for publication bias.

### 3.6. (ATV vs. SMV vs. RSV vs. Placebo): Network Meta-Analysis

Group ‘A’: placebo

Group ‘B’: ATV

Group ‘C’: SMV

Group ‘D’: RSV

#### 3.6.1. Reduction in PD

9 months.

**Figure 13:**
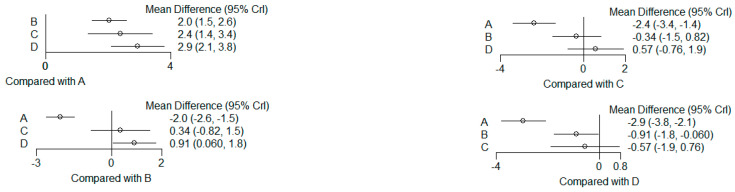
Results of pairwise comparisons from NMA of mean differences of Reduction in PD by Group at 9 months: Relative mean differences and 95% credible interval, I2 overall index of heterogeneity.

#### 3.6.2. Reduction in CAL

9 months.

**Figure 14:**
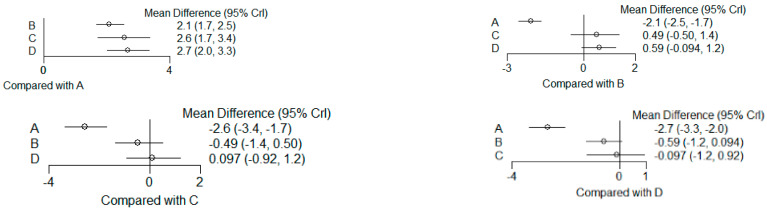
Results of pairwise comparisons from NMA of mean differences of Reduction in CAL by Group at 9 months: Relative mean differences and 95% credible interval, I^2^ overall index of heterogeneity.

#### 3.6.3. Reduction in IBD

9 months.

**Figure 15:**
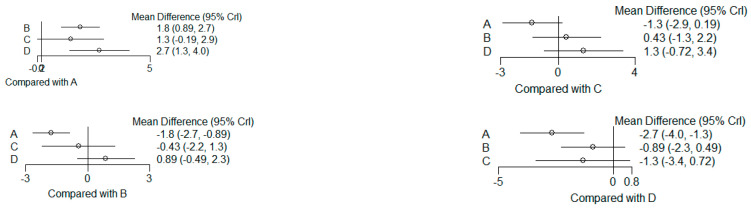
Results of pairwise comparisons from NMA of mean differences of Reduction in IBD by Group at 9 months: Relative mean differences and 95% credible interval, I^2^ overall index of heterogeneity.

#### 3.6.4. Reduction in RX Bone Depth

9 months.

**Figure 16:**
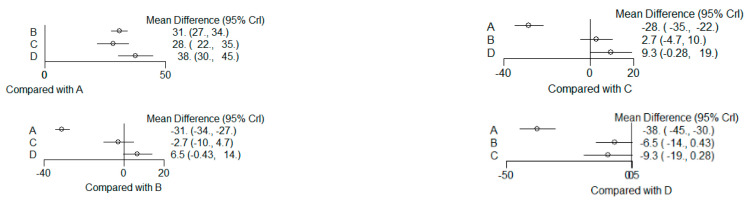
Results of pairwise comparisons from NMA of mean differences of Reduction in Depth by Group at 9 months: Relative mean differences and 95% credible interval, I^2^ overall index of heterogeneity.

## 4. Discussion

The present systematic review has evaluated the effects that the local subgingival delivery of statins has as an adjunct to periodontal disease therapy, based on the assessment of existing RCTs.

Similar results have been observed within the included RCTs regarding the effect on the periodontium, including the reduction in sulcus bleeding and periodontal depth, clinical attachment level gain, and, in the cases where it was analyzed, the impact on the radiographic infrabony defect depth (IBD) in patients with periodontitis [1,2,3,5,6,8,10,11,12,14,18,19,20,21,22,23,24,25].

When comparing the initial evaluation of the parameters to the follow-ups at 3, 6, and up to 9 months, all the studies presented a reduction both in the placebo group and in the statin group. This was also seen when there was a third testing group. This can be explained by the efficacy of the scaling and root planing (SRP) or the open flap debridement (OFD) in combination with SRP in reducing the subgingival periodontal pathogens. Despite the improvement of all these parameters, there were differences between the test statin groups (Simvastatin, Atorvastatin, or Rosuvastatin) and the placebo groups.

Observing the effect of these procedures on the Plaque Index, there was no statistically significant difference between the groups (the placebo and the trial group) in all the studies, except one trial conducted by Vemanaradhya et al. [1,2,3,5,6,8,10,11,12,14,19,20,21,22,23,24,25]. These similar outcomes could be explained by the comparable oral hygiene measures that were undertaken. Before the treatment took place, all patients were provided post-surgical instructions including the proper brushing technique (modified Bass technique).

One parameter analyzed in all the studies was the modified sulcus bleeding index (mSBI). A total of 13 of the studies observed statistical significance in the mSBI between the statin group and the placebo group on the follow-up visits. These results can be justified by the anti-inflammatory effect that the statins were seen to have on the periodontium and the consequent reduction in the modified sulcus bleeding [2,3,6,11,12,14,19,20,21,22,23,24,25]. However, five studies did not present a statistically significant (SS) difference [1,5,8,10,18].

The delivery of SMV as a local drug was practiced [11,18,20,25] on systematically healthy patients. In the study performed by [18], there were statistically significant differences between the placebo and the SMV group when analyzing all parameters except for PD and CAL. This was the only trial that presented only one follow-up at 45 days, which could be the explanation for these insignificant results. The rest of the included trials that used SMV as an adjunctive presented SS differences in all clinical and radiographic parameters between placebo and control groups throughout the follow-ups [11,20,25].

In a study completed by Pradeep et al. [21] where there was the administration of ATV 1.2% in systematically healthy patients, all parameters were statistically significantly higher in the statin group up to 9 months after the subgingival drug administration. In a different study also performed by Pradeep et al. [21], the administration of Rosuvastatin 1.2% resulted in SS higher changes in CAL, PD, and IBD in all follow-up visits, except at 1 month, when the results in CAL were similar between groups. This variable was not comparable with any other study due to the few 1-month follow-ups recorded.

The comparison of these two separate studies with the evaluation of these two different drug administrations (ATV 1.2% and RSV 1.2%) [21,22] demonstrates the higher clinical and radiographic change in the ATV 1.2% group.

On the other hand, two other individual studies [8,24] did their own Randomized Controlled Trial comparing these same two drug administrations to each other and to a placebo group. These results present dissimilarities to the previously mentioned studies, given that both conclude that the statin creating the major positive change to the periodontium was Rosuvastatin.

Comparing SMV 1.2% to ATV 1.2% [21,25] showed that at the 6-month follow-up, the simvastatin group presented higher PD reduction and CAL gain, although IBD was significantly higher in the atorvastatin group.

Analyzing the results from the simvastatin group and the rosuvastatin group from two different trials [24,25], all clinical and radiographic parameters were significantly higher in the Simvastatin group.

To study the effects of statins on the periodontium, a selection of studies compared this type of drug to other drugs that were not hypocholesteromiants, including alendronate (ALN) and metformin (MTF), as well as statin in combination with photodynamic therapy (PDT).

According to Pradeep et al. [1], statins can act as a powerful inhibitor of bone resorption and they have been demonstrated to increase alveolar bone density and decrease bone loss. In this study, an ALN 1% group was compared to an ATV 1.2% group and a placebo group. Both ALN and ATV presented SS higher changes in clinical and radiographic parameters when compared to the placebo group. The evaluation of both medications presented diverse results: while CAL gain was seen to be SS higher in the ATV group, PD, IBD depth, and radiographic DDR% obtained SS more positive results in the Alendronate group.

MTF is a hypoglycemic agent that also has been seen to present anti-inflammatory agents. In a randomized controlled clinical trial conducted by Pankaj et al. [2] RSV 1.2% was delivered in one group and 1% metformin in another group. Both drug deliveries showed SS better results (PD, CAL, and IB) when compared to the placebo group. The comparison between both drug groups showed no statistically significant differences in clinical and radiographic parameters.

Photodynamic therapy was used in a trial conducted by Rahman et al. [10]. The aim of the study was to compare the efficacy of antimicrobial photodynamical therapy to Simvastatin 1.2% combined with scaling and root planing (SRP). At a 3-month follow-up, there was a PD and RAL reduction in the three groups. A higher reduction was observed in the statin group in comparison to the PDT and the placebo groups, although the results were not SS.

The selected trials were not only performed on systematically healthy patients, as studies were also undertaken on two of the main periodontitis risk factor patients: smokers and diabetics. Two selected studies were performed on patients who had been smokers for at least 10 years. The first trial, whose author was Kumari et al. [14], studied the efficacy of Atorvastatin 1.2% on this type of patient. All parameters presented statistically significant differences between the statin group and the placebo group, being significantly higher in the ATV group. The second trial, conducted by Pradeep et al., used Simvastatin as an LDD and also presented advantageous results compared to the placebo group. The comparison of both types of statins used in these previously mentioned trials showed comparable results between each other on the smoking participants [3].

Two RCTs [6,19] were carried out on well-controlled type 2 diabetes patients. In both cases, the statin group presented SS higher changes in all clinical and radiographic parameters at 3, 6, and 9 months. Although results were generally higher in the Simvastatin group in comparison to the ATV group in DM 2 patients, the results were not significant.

The comparison of results in the use of SMV as an adjunct to periodontal therapy between healthy, diabetic, and smoking patients was made in four articles [3,6,20,25]. The variables studied were PD, CAL, and IBD. PD reduction and CAL gain were SS higher in the trials performed on healthy patients [20,25]. In the case of IBD, the results were also observed to be higher in healthy patients [25], although the results were very similar to the study performed on DM patients [6]. This leads us to believe that if the patient presents with systematically controlled diabetes, the results can still be favorable. Through this former comparison, we can state that the smokers were the group that presented the lowest improvements in comparison to the rest of the studies.

While the periodontal depth parameter presented a higher reduction in patients with controlled DM type 2 [19], the rest of the parameters were observed to be higher in the non-diabetic patients [21] when administering ATV in both trials. The delivery of ATV in smokers [14] compared to non-smokers in [21] presented SS higher changes in the non-smoker group.

The addition of Platelet-Rich Fibrin (PRF) to the statin delivery was carried out in three other RCTs [5,12,23]. The first trial compared a placebo group to a PRF group and a PRF and ATV 1.2% group. There were no statistically significant differences in the clinical parameters in the PRF and PRV + ATV groups, although there were SS higher changes in the radiographic parameters in the PRF + ATV group. In another article, where RSV 1.2% was administered instead of ATV, at 9 months there was a SS higher change in the statin + PRF group for PD, CAL, and DDR.

The supplement of PRF compared with RSV alone showed improvement in the clinical and radiographic parameters. The comparison of PRF + ATV and ATV alone also presented SS higher changes in clinical and radiographic parameters when using PRF in addition to the statin [5,24].

A third article used Rosuvastatin 1.2% + PRF + HA. The addition of hydroxyapatite did not improve the results in PD, CAL, or IBD when comparing it to a trial where there was no HA used [5,23].

PRF was added after the SRP and OFD techniques and combined with the post-treatment of antibiotics. The antibiotics used consisted of 500 mg of Amoxicillin for 5 days and 800 mg of Ibuprofen. To evaluate the influence of the technique used and the antibiotics administered post-surgery, a comparison of the placebo groups was conducted. The three studies that used this method did not have superior differences in comparison to the placebo groups of studies that did not use OFD and antibiotics. These comparable results lead us to think that the technique used and the use of antibiotics did not influence the parameters [5,12,23,26,27,28,29,30].

The last variable that was taken into consideration after analyzing all the included articles was how the patients responded to the administered drug. A total of 100% of the patients tolerated the drug, with no adverse reactions, no discomfort, and no post-application complications.

The current systematic review presents some limitations, including the need for more recent RCTs given that the latest one was in 2018. The ROBINS-2 tool shows that over 66% of the included trials display a risk of bias, which is one of the limitations of this study.

## 5. Conclusions

To conclude, statins have proven to have a positive effect on the periodontium by improving both clinical and radiographic parameters by a considerable margin with no known adverse effects on the patients [30]. It has been observed that the administration of this type of drug presents equally positive outcomes on patients with type II diabetes, although the results are not comparable in smokers. Regarding the PD reduction, the Simvastatin group showed a significantly higher reduction than the Atorvastatin group at 6 months. Moreover, the Simvastatin group showed a significantly higher reduction than the Atorvastatin group at 9 months. Finally, no differences between statins were found in the rest of the outcomes.

## Figures and Tables

**Table 1:** Characteristics of included studies. LDD, Local Drug Delivery; SRP, Scaling and Root Planing; GCF, Gingival Crevicular Fluid; IL, Interleukin; SS, Statistical significance; PI, Plaque Index; GI, Gingival Index; SBI, Sulcus Bleeding Index; PPD, Periodonal Probing Depth; CAL, Clinical Attachment Level; mSBI, modified Sulcus Bleeding Index; ATV, Atorvastatin; CP, Chronic Periodontitis; IBD, Infrabony depth; AL, Alendronate; RSV, Rosuvastatin; MF, Metformin; DM2, Diabetes Mellitus 2.

	Aim of Study	Type of Drug and Dose	Follow-Ups	Sample	Outcomes	Conclusions
Author Year Journal Reference				Sample Number Sample Ages Other Sample Characteristics	Patients That Finished the Study Adverse Effects of the Drug Clinical Parameters Radiographic Parameters	
Vemanaradhya et al., 2017 *Archives of Oral Biology* [18]	Evaluate the efficacy of 1.2% simvastatin gel as an LDD in adjunct SRP on GCF IL-6 and IL-8 levels in chronic periodontitis patients and correlate their values with clinical parameters	Simvastatin 1.2%	45 days	46 35–60 years old 18 males and 28 females	Acceptable statin toleration Reduction in all clinical parameters in all groups SS higher reduction in statin group for PI, GI, and SBI No SS differences in PPD and CAL between groups	Simvastatin 1.2% has an effective role in controlling the inflammation of the periodontium.
Kumari et al., 2016 *Journal of Investigative and Clinical Dentistry* [14]	Evaluate the efficacy of a 1.2% ATV local drug delivery as an adjunct to SRP for the treatment of IBD (intrabody defects) in smokers with CP in comparison with placebo gel.	Atorvastatin 1.2%	3–6–9 months	71 smokers 30–50 years old	66 Acceptable statin toleration SS higher mSBI and PD reduction and CAL gain in statin group SS higher reduction in IBD in statin group	Significant improvement in clinical parameters compared to placebo gel as an adjunct to SRP.
Pradeep et al., 2016 *Journal of Investigative and Clinical Dentistry* [1]	Evaluate and compare the efficacy of 1% ALN and 1.2% ATV gel as a local drug delivery system in adjunct to scaling and root planing (SRP) for treatment for intrabony defects in CP patients.	Atorvastatin 1.2% Alendronate 1%	3–6–9 months	104 30–50 years old 53 males and 51 females	90 Acceptable statin toleration No SS differences in PI between groups. ALD and ATV groups showed SS in all parameters. ALN showed SS differences in PD, CAL, and DDR% when compared to ATV.	Both ATV and ALN can be used as an effective mode of treatment for CP patients. However, ALN was comparatively better than ATV.
Kumari et al., 2016 *Journal of Periodontology* [19]	Evaluate the effectiveness of 1.2 ATV gel, as an adjunct to SRP in the treatment of infrabony defects in chronic periodontitis in subjects with DM2	1.2% Atorvastatin	3–6–9 months	75 individuals 40–50 years old. 38 males and 37 females	60 Acceptable statin toleration SS greater mSBI and PD reduction, RAL gain, and IBD reduction in statin group.	Local delivery of 1.2% ATV into periodontal pockets of type 2 DM patients stimulated a significant improvement in clinical and rx parameters as compared to placebo gel
Pankaj et al., 2018 *Journal of Periodontology* [2]	Investigate the effectiveness (both clinical and rx) of locally delivered 1.2% RSV gel, 1% MF gel, and placebo as an adjunct to SRP in the treatment of infrabony defects.	1.2% Rosuvastatin 1% Metrformin	6–12 months	90 patients 25 to 45 years old 44 males and 46 females	Acceptable statin toleration Both groups displayed improvement in PI and mSBI, but no SS difference in PI between groups. RSV and MF higher decrease in mSBI, PD, and CAL values and also greater depth reduction (radiographically) and higher DDR% Comparing RSV and MF, RSV showed statistically significant improvement in PD and CAL and also greater DDR%	The study shows that both drugs are used as an affective mode of treatment. However, RSV was comparatively better than MF.
Martande et al., 2016 *Journal of Periodontology* [12]	Evaluate the combined efficacy of PRF and 1.2% ATV gel with OFD in treatment of infrabony defects in CP individuals RCCT	1.2% Atorvastatin Platelet Rich Fibrin	9 months	96 patients mean age of 37.6 48 males and 48 females	90 Acceptable statin toleration SS reduction in PI and mSBI in all three groups Greater PD reduction and RAL gain in PRF and PRF + ATV sites with no SS differences between them.	There was a greater improvement in clinical parameters in PRF + ATV and PRF as compared to the control group. 1.2% ATV failed to increase the regenerative potential by PRF alone due to similar outcomes between the trial groups.
Pradeep et al., 2016 *Journal of Periodontology* [8]	Investigate the clinical and rx effects of LDD and re-delivery of 1.2% RSV and 1.2% ATV gels with scaling and root planing in the treatment of 2/3-welled IBD in CP patients.	1.2% Rosuvastatin 1.2% Atrovastatin	6–9 months	90 patients aged 25 to 45 45 males and 45 females	81 Acceptable statin toleration Reduction in PI, mSBI, and PD as well as CA gain was observed in all groups, except PI reduction in group 1. DDR was SS in the statin groups. No SS difference in the mean PI and mSBI reduction between both control groups between baseline at 3, 6, and 9. However, PI reduction between 6 and 9 m, PD reduction, CA level gain, and DDR were significantly greater in RSV than ATV from BL-6 and 6–9.	The administration of statins is superior to mechanical periodontal therapy alone, with LDD of 1.2% rosuvastatin resulting in significantly greater clinic-radiographic improvements compared to 1.2% ATV.
Pradeep et al., 2016 *Journal of Periodontology* [5]	Investigate the clinico-rx effects of OFD, OFD + PRF, and OFD + PRF + 1.2% RSV in the treatment of 2/3-walled IBD in CP patients.	1.2% Rosuvastatin Platelet Rich Fibrin	9 months	90 patients 25–45 years old	90 Acceptable statin toleration All groups showed significant improvements in all periodontal outcomes from BL-9 months. No SS differences between the 3 groups for mSBI and PI PD and IBD depth reductions as well as CA level gain were significantly greater in control groups. Significantly greater improvements in these parameters were found in group 3 over 2.	The use of RSV has a positive effect on the parameters.
Priyanka et al., 2017 *The International Journal of Periodontics & Restorative Dentistry* [11]	Investigate the effectiveness of 1.2 mg SMV as a local drug delivery system and as an adjunct to scaling and root planing in the treatment of aggressive periodontitis	1.2% Simvastatin	3–6 months	24 patients 30 to 50 years old 14 males and 10 females	21 Acceptable statin toleration PI, both groups maintained comparable levels of oral hygiene. mSBI presented a greater reduction in the SMV group PD, greater reduction in the SMV group CAL, greater gain in group 2. Bone fill is also greater in group 2.	This clinical trial demonstrates that local delivery of 1.2 mg of simvastatin into the periodontal pocket in the group 2 patients stimulated a significant increase in PD reduction and CAL gain, and improved bone fill compared to group 1 patients.
Rao et al., 2013 *Australian Dental Journal* [3]	Evaluate the efficacy of SMV 1% as local drug delivery as an adjunct to SRP for the treatment of smokers with CP in comparison with placebo gel	1.2% Simvastatin	3–6–9 months	40 male smokers 30–50 years old.	35 Acceptable statin toleration Both groups presented an improvement in PI but no SS in both parameters at any visit. Significant decrease in mSBI in the SMV group compared to placebo. SV greater CAL gain and PD decrease in the SMV group. SMV group showed SS IBD mean reduction. SS greater vertical bone defect fill in SMV group.	LDD of 1.2 5 SMV in smokers stimulated a significant increase in PD reduction, CAL gain, and improved bone fill as compared to placebo gel as an adjunct to SRP.
Rahman et al., 2017 *Photodiagnosis and Photodynamic Therapy* [10]	Evaluate and compare the efficacy of antimicrobial photodynamic therapy and the LDD of 1.2% SMV gel as an adjunct to SRP versus SRP alone in the treatment of periodontitis using clinical, microbiological, and biochemical parameters	1.2% Simvastatin	3 months	15 8 males and females 35–60 years old	Acceptable statin toleration Significant reduction in API, PBI, PPD, and RAL in all three groups. The mean PPD and RAL score reduction was found to be higher in group 3 (SMV), with no SS.	All three treatment groups showed similar efficacy in improving clinical parameters and reducing p-gingivalis and GCF RANKL levels with no SS difference in outcomes. Between both adjunct treatments used, neither has been proven to have greater potential than the other.
Pradeep et al., 2012 *Journal of Periodontology* [20]	Investigate the clinical and radiographic efficacy (bone fill) of 1.2 mg SMV as an adjunct to SRP in the treatment of mandibular buccal class II furcation defects.	1.2% Simvastatin	3–6 months	72 38 males and 34 females 30–50 years old	Acceptable statin toleration. Decrease in mSBI was greater in the SMV group PD: there were statistical differences in the decrease, being greater in group 2. RVAL and RHAL: greater in group of SMV Bone fill: also greater in group 2	There was a greater decrease in gingival index and PD and more RVAL and RHAL gain with significant bone fill with locally delivered SMV in class II furcation defects
Pradeep et al., 2012 *Journal of Periodontology* [6]	Evaluate the efficacy of 1.2% SMV as an LDD in adjunct to SRP for the treatment of patients with DM2 and CP compared to placebo gel.	1.2% Simvastatin	6–9 months	38 20 males and 18 females 30–50 years old	35 Acceptable statin toleration. No SS difference in PI. SS decreased in the SMV group for mSBI. SMV group presented greater PD reduction, CAL gain, IBD, and vertical defect fill.	The local delivery of 1.2% SMV into periodontal pockets of patients with type 2 diabetes stimulated a significant increase in PD reduction, CAL gain, and improved bone fill compared to placebo gel as an adjunct to SRP.
Pradeep et al., 2013 *Journal of Periodontology* [21]	Evaluate the efficacy of 1.2% ATV as local drug delivery in comparison with placebo gel in adjunct to SRP for the treatment of intrabony defects in individuals with CP.	1.2% Atorvastatin	3–6–9 months	67 35 males and 32 females 30–50 years old	60 Both groups showed improvement in PI. Decrease in mSBI, PD reduction, and CAL gain in the ATV group. ATV group showed significantly greater vertical radiographic defect fill.	The local delivery of 1.2% ATV in individuals with CP stimulates a significant increase in PD reduction, CAL gain, and improved bone fill in adjunct to SRP compared to placebo gel.
Pradeep et al., 2015 *Journal of Periodontology* [22]	Investigate the clinical and radiographic effects of locally delivered 1.2% RSV gel as an adjunct to the non-surgical treatment in CP patients when compared to placebo gel.	1.2% Rosuvastatin	1–3–4 and 6 months	70 individuals 25–55 years old	65 Acceptable statin toleration. Higher decrease in mSBI in RSV group RSV. CAL and PD SS RSV group. No SS in PI. Greater bone fills in RSV group	The local delivery of RSV reduced inflammation and induced bone formation.
Pradeep et al., 2016 *Journal of Periodontology* [23]	Evaluate the potency of a combination of RSV 1.2 mg in situ gel with 1:1 mixture of autologous PRF and HA bone graft in the surgical treatment of mandibular degree II furcation defects when compared with autologous PRF and A bone graft placed after OFD	1.2% Rosuvastatin Autologous platelet-rich fibrin Porus hydroxyappatite bone graft.	9 months	110 60 males and 50 females 25–55 years old	105 Acceptable statin toleration SS in RSV group when looking at mSBI and PI SS in the group’s parameters at follow-up. The groups with RSV presented SS better changes in all parameters (PD, RVAL, RHAL, and IBD).	The addition of RSV to the PRF and HA improved the regenerative effect on the bone defects.
Garg et al., 2017 *Journal of Periodontology* [24]	Explore the efficacy of 1.2% RSV and 1.2% ATV gel as a local drug delivery and redelivery system as an adjunct to SRP for treatment of class II furcation defects.	1.2% Rosuvastain 1.2% Atorvastatin	6–9 months	90 (55 males and 60 females)	90 Acceptable statin toleration SS differences in mSBI in RSV in comparison to ATV PD, RHCAL, and RVCAL, SS higher changes in the statin groups, although it was higher in RSV. Bone depth reduction is greater in statin groups.	RSV has greater anti-inflammatory effects due to more effective CRP levels
Pradeep et al., 2010 *Journal of Peritology* [25]	Investigate the clinical and radiologic efficacy of SMV, 1.2 mg as an adjunct to SRP in the treatment of CP.	1.2% Simvastatin	1–2–4–6 months	64	60 Acceptable statin toleration. mSBI decrease in both groups, although greater in SMV group. PD, CAL, and IBS were SS in SMV group.	Greater decrease in clinical and radiographic parameters in Simvastatin patients.
